# Magnetic resonance imaging of human variegated squirrel bornavirus 1 (VSBV-1) encephalitis reveals diagnostic pattern indistinguishable from Borna disease virus 1 (BoDV-1) encephalitis but typical for bornaviruses

**DOI:** 10.1080/22221751.2023.2179348

**Published:** 2023-02-27

**Authors:** Monika Huhndorf, Julia Juhasz, Mike P. Wattjes, Andreas Schilling, Stefan Schob, Ingmar Kaden, Günter Klaß, Dennis Tappe

**Affiliations:** a Clinic of Radiology and Neuroradiology, University Hospital Schleswig-Holstein, Kiel, Germany; b Department of Neuroradiology, University Medical Center Göttingen, Göttingen, Germany; c Institut für diagnostische und interventionelle Neuroradiologie, Medizinische Hochschule Hannover, Hannover, Germany; d Klinikum Frankfurt/Oder, Frankfurt (Oder), Germany; e Universitätsklinik und Poliklinik für Radiologie Halle, Halle (Saale), Germany; f BG Klinikum Bergmannstrost, Halle (Saale), Germany; g Mathias-Spital Rheine, Rheine, Germany; h Bernhard Nocht Institute for Tropical Medicine, Hamburg, Germany

**Keywords:** Bornavirus, variegated squirrel bornavirus 1, VSBV-1, Borna disease virus 1, BoDV-1, encephalitis, basal ganglia, limbic system

## Abstract

Human bornavirus encephalitis is an emerging disease caused by the variegated squirrel bornavirus 1 (VSBV-1) and the Borna disease virus 1 (BoDV-1). While characteristic brain magnetic resonance imaging (MRI) changes have been described for BoDV-1 encephalitis, only scarce diagnostic data in VSBV-1 encephalitis exist. We systematically analysed brain MRI scans from all known VSBV-1 encephalitis patients. Initial and follow-up scans demonstrated characteristic T2 hyperintense lesions in the limbic system and the basal ganglia, followed by the brainstem. No involvement of the cerebellar cortex was seen. Deep white matter affection occurred in a later stage of the disease. Strict symmetry of pathologic changes was seen in 62%. T2 hyperintense areas were often associated with low T1 signal intensity and with mass effect. Sinusitis in three patients on the first MRI and an early involvement of the limbic system suggest an olfactory route of VSBV-1 entry. The viral spread could occur *per continuitatem* to adjacent anatomical brain regions or along specific neural tracts to more distant brain regions. The number and extent of lesions did not correlate with the length of patients’ survivals. The overall pattern closely resembles that described for BoDV-1 encephalitis. The exact bornavirus species can thus not be deduced from imaging results alone, and molecular testing and serology should be performed to confirm the causative bornavirus. As VSBV-1 is likely of tropical origin, and MRI investigations are increasingly available globally, imaging techniques might be helpful to facilitate an early presumptive diagnosis of VSBV-1 encephalitis when molecular and/or serological testing is not available.

## Introduction

Human bornavirus encephalitis is a severe zoonotic disease caused by two closely related members of the *Bornaviridae* family, the variegated squirrel bornavirus 1 (VSBV-1; species *Mammalian 2 orthobornavirus*) and the Borna disease virus 1 (BoDV-1; species *Mammalian 1 orthobornavirus*). While VSBV-1 is acquired through contact to exotic squirrels in private holdings and zoological gardens [1–6], BoDV-1 is harboured by the bicoloured white-toothed shrew in virus-endemic regions of Germany, Austria, Switzerland and Liechtenstein [7]. VSBV-1 has been discovered in 2015 retrospectively in infected private exotic squirrel breeders in Germany [1], whereas BoDV-1 caused encephalitis in humans in 2018 in Germany [8,9]. Human bornavirus encephalitis caused by either of these viruses has a high case-fatality rate [3,4,10], and the course of BoDV-1 encephalitis seems to be more rapid than the course of VSBV-1 encephalitis [6,11].

As of December 2022, a total of five confirmed and two possible fatal VSBV-1 encephalitis cases have been recorded in private exotic squirrel breeders and zoo animal caretakers in Germany [1–6], contrasting the case count of fatal BoDV-1 encephalitis which currently exceeds 40 proven sporadic cases in Germany ([6,8,9,11–15]; and unpublished cases). Case definitions and diagnostic laboratory testing schemes [6], as well as neuropathological features have been published for both BoDV-1 and VSBV-1 encephalitis [13,16]. However, while a characteristic indicative cranial magnetic resonance imaging (MRI) pattern has been described recently for BoDV-1 encephalitis in a systematic study [17], data on brain MRI characteristics in human VSBV-1 encephalitis are very limited.

The aim of this study was to analyse brain MRI scans of all known VSBV-1 encephalitis cases in a standardized fashion in order to identify typical imaging patterns and secondly compare these patterns with the described radiological BoDV-1 phenotype, potentially facilitating an early and accurate diagnosis of affected patients. For those patients with follow-up imaging studies available, we aimed to describe the lesion evolution of VSBV-1 brain manifestations in the context of the clinical presentation.

## Patients and methods

We retrospectively analysed a total of 26 MRI brain scans obtained from five proven and two possible cases of VSBV-1 encephalitis according to the case definition [6] with eventually fatal outcome (Table 1) in parallel to the respective patient documentation. Brain MRI follow-up studies were available in six patients. Ethical clearance for this study was obtained from the local ethics committee (Medical Board of Hamburg, no. PV5616).

**Table 1.** Clinical characteristics of seven patients with fatal VSBV-1 encephalitis included in the study.

Patient Code* (Alternative Code)	Year of Infection	Age at Infection	Sex	Duration of Disease	Status of VSBV-1 Infection (Material)	VSBV-1 Serostatus around Time of Death***	Exotic Squirrel Contact	Reference
A (3**)	2013	72	M	4 months	Confirmed (Autopsy)	Seropositive (1:5,120 in serum and 1:2,560 in CSF)	Breeder	(1,3,4,16)
B (2**)	2013	62	M	2 months	Confirmed (Autopsy)	No serum or CSF available	Breeder	(1,3,4,16)
C (1**)	2011	63	M	3 months	Confirmed (Biopsy)	No serum or CSF available	Breeder	(1,3,4,16)
**D1**	2007	29	M	12 years	Confirmed (Biopsy)	Seropositive (1:655,360 in serum and 1:20,480 in CSF)	Zoo Animal Caretaker	(4,6)
D2 (4**)	2013	45	F	3 months	Confirmed (Autopsy)	Seropositive (1:2,560 CSF; no serum available)	Zoo Animal Caretaker	(2,3,4,16)
**F**	2005	65	M	12 months	Possible (no biopsy or autopsy performed)	No serum or CSF available	Breeder	(3,4)
**G**	2006	75	M	1 month	Possible (autopsy material no longer available)	No serum or CSF available	Breeder	(3,4)

Notes: * A probable case, case E (3,4), was not included in this study as no cranial magnetic resonance imaging had been performed. **This alternative code has been used in (1,16). *** As determined by IgG immunofluorescence antibody test (IFAT). CSF, cerebrospinal fluid.

The virus was detected in the five patients with confirmed VSBV-1 encephalitis (cases A, B, C, D1 and D2) in brain tissue by polymerase chain reaction (PCR), *in situ* hybridization and/or immunohistochemistry [1,2,6]. In two patients with possible infection (cases F and G), encephalitis without evidence for other causative pathogens combined with epidemiological links to infected exotic squirrels was present, but no autopsy material was available for confirmation [3,4].

Similar to the regions defined for evaluation of MR imaging in BoDV-1 encephalitis [17] 30 brain regions were defined (Table 2) and evaluated by two neuroradiologists (M.H. and J.J.) with 9–10 years of experience in this VSBV-1 series here. Evaluation encompassed presence or absence of abnormal imaging findings on T1 and T2 weighted/fluid-attenuated inversion recovery (FLAIR) images, diffusion-weighted imaging (DWI), and contrast enhancement on contrast-enhanced T1 weighted images, regarding disturbance of blood-brain barrier as well as pachy- and leptomeningeal enhancement. Abnormal imaging signal intensities were characterized regarding delineation and mass effect. Inconclusive image interpretation results between both readers were discussed until a consensus agreement was achieved. In the second step, the temporal evolution of MRI was analysed for each patient individually. Duration until first imaging changes occurred differed markedly between all patients, as well as the frequency of MRI scans during the course of disease. To avoid bias in the assessment of the evolvement of pathological findings, MR scans of all patients were analysed in a random order and no more than 5 scans were analysed in one session with the maximum of one session per week. Finally, we compared our MR findings of VSBV-1 encephalitis patients with the already published imaging phenotype of BoDV-1 encephalitis [17].

**Table 2.** Pathological changes in at least one brain MR examination in seven VSBV-1 encephalitis patients.

Brain region	Number of patients in whom the following regions were affected on MR imaging
Hippocampus	7
Caudate nucleus	7
Mesencephalon	6
Putamen	6
Amygdala	6
Pons	5
Deep WM frontal	5
GM insular	5
Medulla oblongata	4
Globus pallidus	4
External capsule	4
Corpus callosum	4
Deep WM temporal	4
Deep WM parietal	4
Subcortical WM frontal	4
Subcortical WM temporal	4
GM frontal	4
Cingulate gyrus	4
Dentate nucleus	3
Cerebellar WM	3
Thalamus	3
Internal capsule	3
Deep WM occipital	3
GM temporal	3
Subcortical WM parietal	2
GM parietal	2
Subcortical WM occipital	1
GM occipital	1
Cerebellar GM	0
Vermis	0

WM: white matter; GM: cortical grey matter.

## Results

Owing to the retrospective nature of our analysis and the multicentric data collection from different secondary and tertiary hospitals, MR image acquisition protocols differed regarding pulse sequences, administration of contrast agents, spatial resolution (in-plane and slice thickness ranging from 2 to 6 mm), scan orientation and repositioning. Twenty-five scans were performed at 1.5 Tesla (T), while one scan was performed at 3 T. The 3 T scan was the first MRI scan of patient D2, in which no signal abnormalities were seen so that the difference in image quality between 3 and 1.5 T did not influence further assessment of the time course.

T2 weighted and/or FLAIR images were acquired in all 26 MR examinations and were both used to assess T2 signal changes. T1 weighted images without contrast agents were acquired in 25 examinations, while T1 weighted images with contrast agents were acquired in 22 examinations. DWI was performed in 22 examinations.

**Figure 1.**
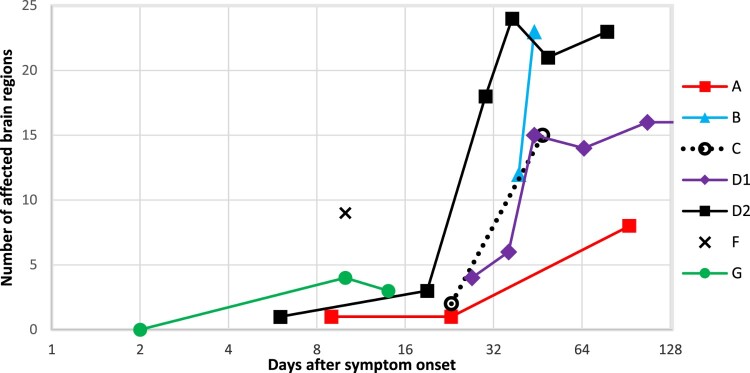
Number of affected brain regions on MR examination per patient and day after symptom onset. The number of affected regions increases markedly over time, well visible on the logarithmic scale. Patient G had a normal MR scan on day 2. Patient D1 had multiple follow-up MR examinations without further change in the number of affected brain regions (not shown). Patient F had one MR scan only. See also Table 3.

The time interval between symptom onset and first brain MRI ranged from 2 to 39 days (median = 10). Time interval between symptom onset and death ranged from 25 days to almost 12 years (median = 86 days). Baseline MR examination was normal in one of the seven patients (patient G on day two), followed by few pathologic findings on day six (patient D2), day 9 (patient A), day 23 (patient C) and day 27 (patient D1); for details see Table 3. Thus, the shortest period between symptom onset and detectable pathologic changes on MRI was six days. The number of affected brain areas increased in all patients over time in whom more than one MR examination was performed and seemed to reach a plateau in patients with more than five MR examinations (Figure 1). There was no relationship between the extent and number of lesions and the patients’ survival. However, a relationship between the height of the antibody titre in serum and cerebrospinal fluid (when available) with the individual duration of disease became apparent.

**Table 3.** Simplified summary of positive magnetic resonance T2 signal changes in anatomical brain regions per patient over time.

	MR results days after symptom onset						
Patient	0–10	11–20	21–30	31–40	41–50	51–80	>81
A	d 9 vermian WM	N.D.	d 23 limbic system vermian WM	N.D.	N.D.	N.D.	d 93 basal ganglia, deep WM, cerebellar WM
B	N.D.	N.D.	N.D.	d 39 limbic system, basal ganglia, external capsule, corpus callosum, GM	d 44 thalamus, mesencephalon, pons, medulla oblongata, internal capsule, deep WM; *basal ganglia (T1)*	no further MR examination	
C	N.D.	N.D.	d 23 limbic system, insulary GM	N.D.	d 47 basal ganglia, thalamus, mesencephalon, pons, external capsule, corpus callosum, deep WM, GM	no further MR examination	
D1	N.D.	N.D.	d 27 limbic system, basal ganglia	d 36 insulary GM, deep WM	d 44 mesencephalon, pons, medulla oblongata, internal/external capsule, corpus callosum, GM, dentate nucleus	d 65 deep WM; corpus callosum dentate nucleaus *basal ganglia (T1)*	d 107 extended deep WM lesions, cerebellar WM; basal ganglia, mesencephalon, medulla oblongata
D2	d 6 limbic system	d 19 basal ganglia, corpus callosum; limbic system *basal ganglia (T1)*	d 30 limbic system, thalamus, mesencephalon, pons, medulla oblongata, internal/external capsule, deep/subcortical WM, GM	d 37 dentate nucleus, cerebellar WM	d 49 *limbic system (T1)*	d 78 *focal cortical and callosal hyperintensity (T1)*	no further MR examination
F	d 10 limbic system, basal ganglia, insulary GM, mesencephalon, pons, medulla oblongata	no further MR examination					
G	d 10 limbic system, basal ganglia, mesencephalon	d 14 mesencephalon	no further MR examination				

Notes: Specific parts of the limbic system, the basal ganglia and regions of deep and subcortical white matter (WM) and cortical grey matter (GM) are not listed in detail. The MR signal of anatomical regions that had normalized during the respective follow-up MR are written in crossed-out letters; otherwise previous changes persisted. T1 signal increase is documented in italics. Negative imaging results were obtained only for case G after 2 days. N.D., not done.

**Figure 2.**
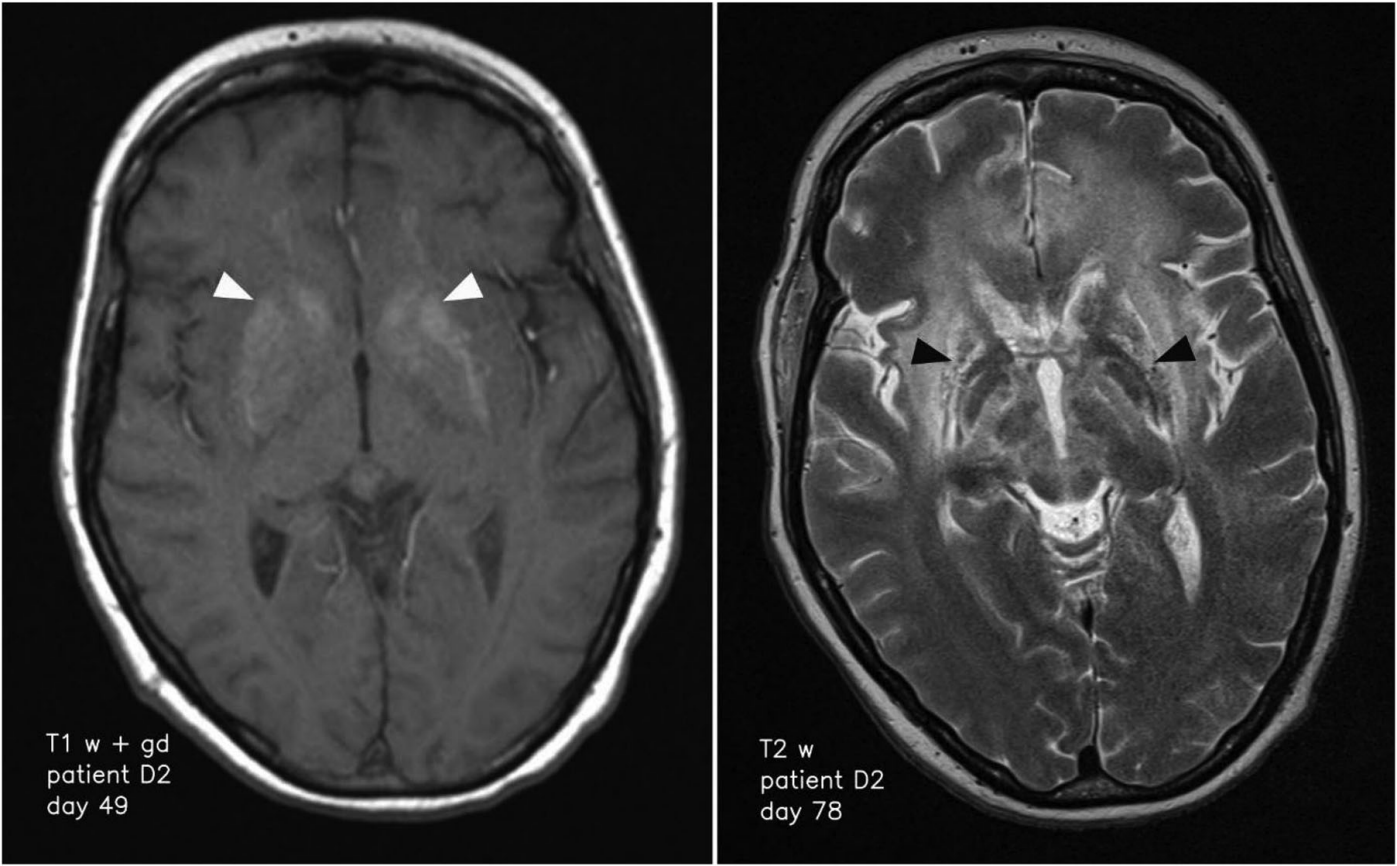
Transformation of the basal ganglia and respective changes on MR imaging over time. High signal of basal ganglia on T1 weighted imaging on day 49 after symptom onset (left; white arrowheads) and structural damage to the basal ganglia 72 days after symptom onset (right; black arrowheads).

**Figure 3.**
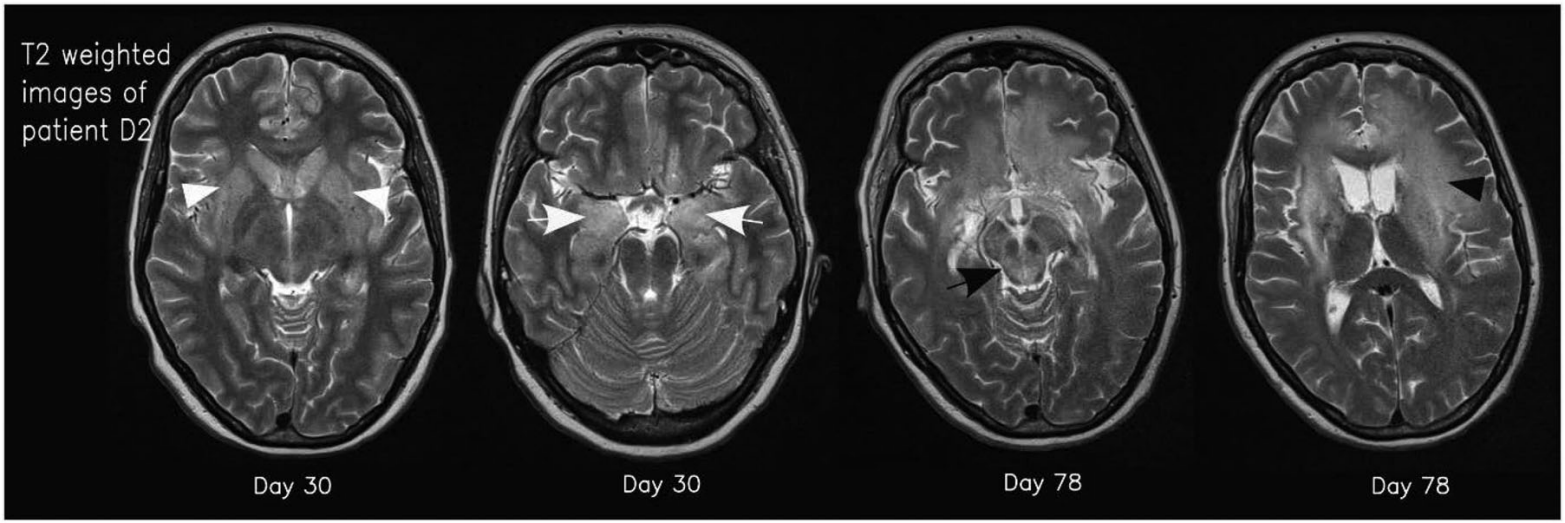
Axial T2 weighted images of patient D2 on days 30 and 78. Typical signal increase of the basal ganglia (white arrowheads) and the amygdala/hippocampus (white arrows) as well as hyperintensity in the mesencephalon (black arrow) and white matter (black arrowhead) is visible.

Most pathologic MRI changes appeared as increased signal on T2 weighted/FLAIR images. The hippocampus and caudate nucleus showed pathologic high T2 signal intensities in all cases while the cerebellar cortex did not show pathologic changes in any of the patients. All other brain regions showed pathologic high T2 signal intensities in at least one patient (Table 2). Three cases showed high T1 signal intensities of parts of the basal ganglia in follow-up scans without a specified time interval (Figure 2). In detail, patient B showed T1 hyperintensity of the putamen and globus pallidus on day 44, while patients D1 and D2 showed T1 hyperintensity of the putamen and caudate nucleus on day 65 and 19, respectively. In the time course, patients D1 and D2 also presented T1 hyperintensity of the globus pallidus on day 149 and 30, respectively. Patient D2 additionally showed T1 hyperintensity of the amygdala and cingulate gyrus on day 49 and 78, respectively (Table 3). Distinct signal increase on T2 weighted images was often associated with signal decrease on T1 weighted images and mass effect of the corresponding brain region suggestive for oedema. This can especially be observed in the basal ganglia, limbic system and white matter (WM) of patients D1 and D2 (Figure 3). When swelling of the basal ganglia regressed, especially putamen, globus pallidus and caudate nucleus, atrophy of those structures could be visualized (Figure 2). This can also be seen in WM changes in patient D1 approximately one year after symptom onset.

Areas with high signal intensities on the DWI with low apparent diffusion coefficient (ADC) values suggestive of restricted diffusion was seen in three patients (B, D1, D2) in different brain regions between day 19 and 78 after symptom onset. There was no leptomeningeal contrast enhancement. Pachymeningeal contrast enhancement was seen in patient A on day 23. Patient B showed ependymal enhancement encompassing the entire ventricular system on day 44. Ependymal enhancement was also seen in patient D2 on days 49 and 78 in the mesencephalic aqueduct, the forth ventricle and cranial parts of central canal of the cervical spine. Parenchymal contrast enhancement was only seen in patient D2 on day 78 with a moderate, almost homogeneous enhancement of the putamen, globus pallidus, and caudate nucleus, as well as in patient B on day 44 with moderate inhomogeneous segmental cortical enhancement of parts of T2 hyperintense areas biparietally and bitemporally.

**Figure 4.**
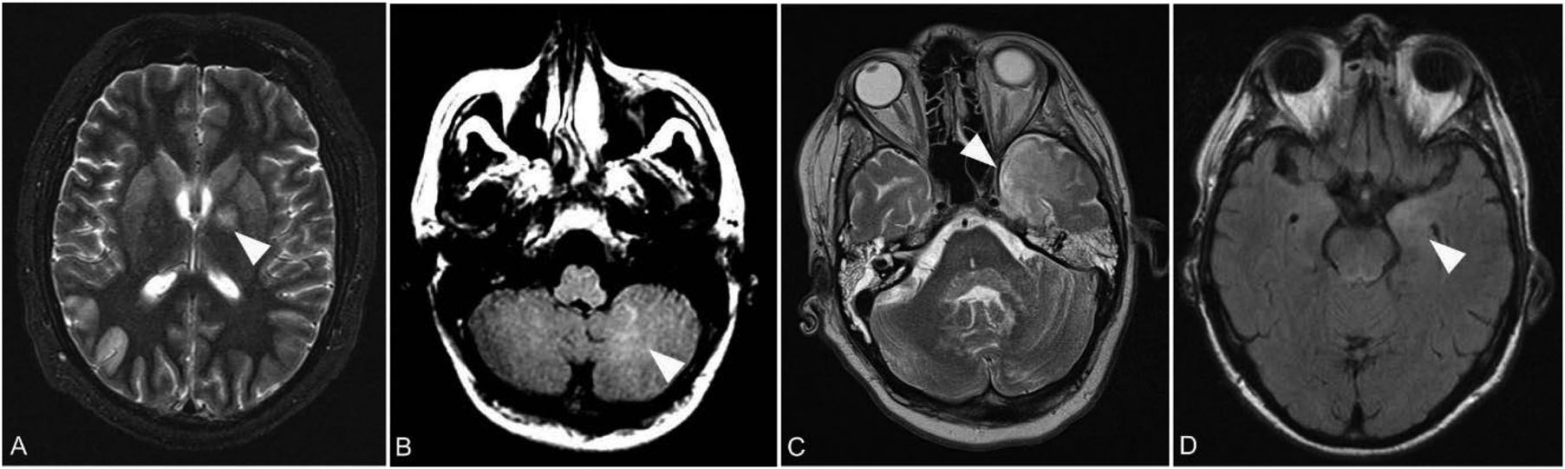
Representative asymmetric lesions. Axial T2 weighted image with left hemispheric changes of the globus pallidus (arrow) in patient B on day 39 (A). Axial FLAIR image with unilateral signal increase of cerebellar white matter (arrow) in patient D1 on day 107 (B). Axial T2 weighted image showing left hemispheric signal increase of the temporal lobe (arrow) in patient D2 on day 78 (C). Axial FLAIR image demonstrating unilateral signal increase of the left amygdala (arrow) in patient F on day 10 (D).

In general, symmetric lesions were noticed. Slight asymmetry of pathologic T2 changes was seen in five of the seven patients without a predominance of certain findings. In detail, patient B showed asymmetric changes of the globus pallidus, frontal and insular cortical grey matter (GM) and the cingulate gyrus on the first scan and of the dentate nucleus und occipital GM 5 days later on the second scan. Patient C showed asymmetric findings of the thalamus. Patient D1 showed asymmetric findings of the cerebellar WM first appearing on scan 5 and then spreading to the contralateral side on scan 6. The external capsule was first affected on the left side at scan 4 while the right side was affected more on at scan 9. Patient D2 showed asymmetric T2 signal changes in the deep frontal WM, temporal and parietal as well as in the frontal and temporal GM. Patient F showed asymmetry of changes of the putamen, caudate nucleus, amygdala and hippocampus. Examples are shown in Figure 4.

**Figure 5.**
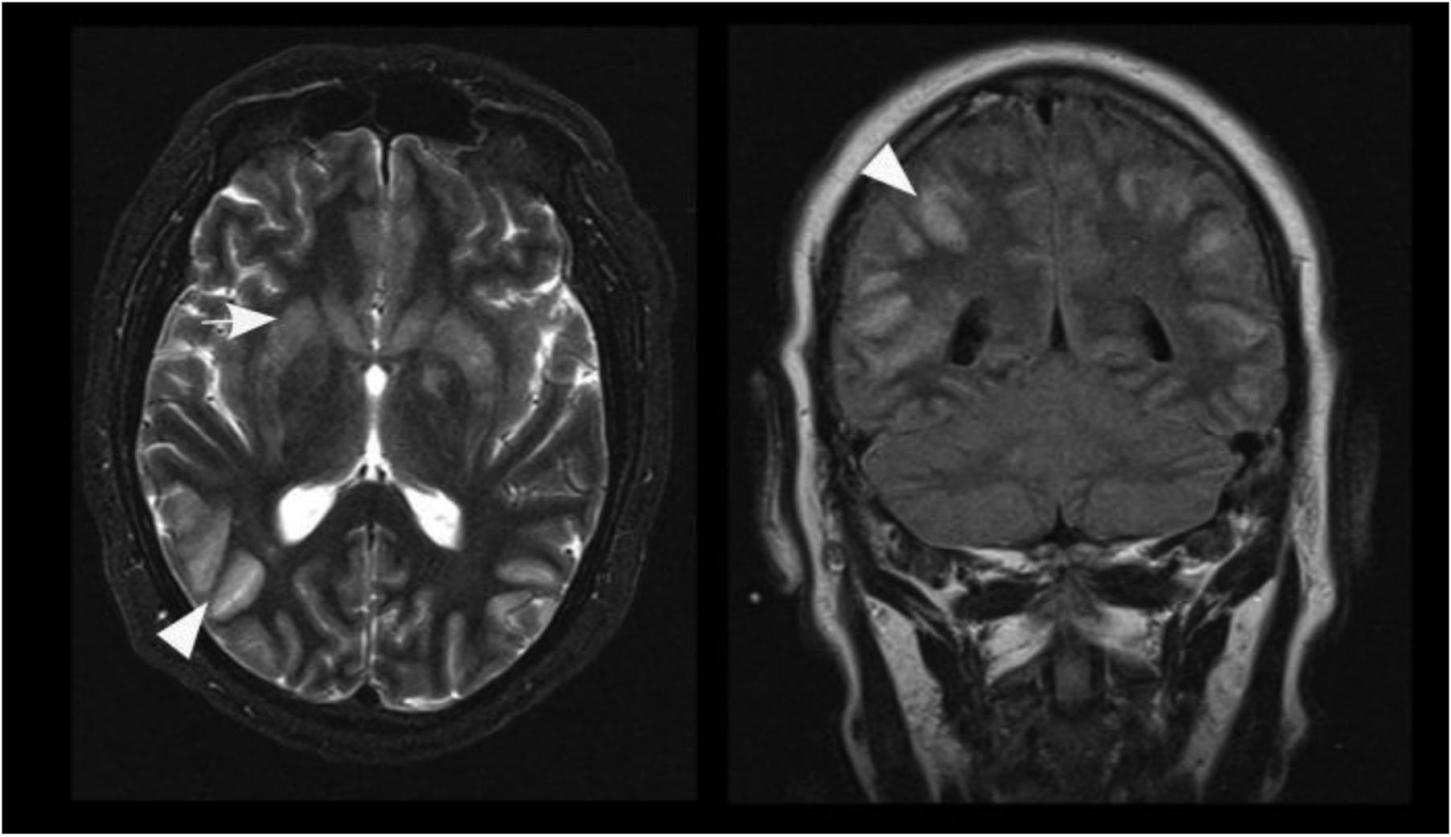
Axial T2 weighted image (left) and coronal FLAIR image (right) of patient B on day 44. Prominent cortical hyperintensities and swelling of cortical grey matter (arrowheads) dominate over signal increase of the basal ganglia (arrow) in this patient.

The time course of signal changes differed within the patient cohort during the development of the disease (Table 3). Parts of the limbic system, especially the hippocampus and the cingulate gyrus, and less frequently the amygdala, showed pathologic signal changes on first suspicious MR examination in all patients. This was mainly followed or accompanied by changes of the basal ganglia (in 7/7 patients) and mesencephalic/pontine/medullary changes (in 6/7 patients). WM involvement was seen in five of the patients and occurred later than day 30 after symptom onset (Figure 3). However, considering changes that were most pronounced, signal intensities changes in the basal ganglia were the leading finding in four of the seven patients (D1, D2, F, G). There were two patients (B and C) in whom T2 signal increase in cortical regions was dominant to changes of the basal ganglia and the limbic system on first MR examination on day 23 and 39, respectively (Figure 5). Patient A showed subtle signal increase in the left hippocampus followed by periventricular WM changes and unilateral signal increase in the head of the caudate nucleus and thus overall discreet findings as far as it can be assessed with an imaging gap of 70 days. Of note, sinusitis was present on first MR examination of patients C (day 23), F and G (day 10 each). In the latter two individuals, sinusitis was clinically apparent during the first medical consultation.

## Discussion

In this study, we analysed brain imaging data from VSBV-1 encephalitis patients by a systematic and standardized approach and compared the findings with the phenotype of BoDV-1 encephalitis. T2 weighted imaging revealed a pattern for VSBV-1 encephalitis which is similar to what was shown for BoDV-1 [17]. However, while DWI as well as contrast enhancement do not seem to be helpful diagnostic markers in VSBV-1 encephalitis, accompanying diffusion restriction of T2 hyperintense lesions was common in BoDV-1 encephalitis [17]. In contrast to findings in BoDV-1 encephalitis, strict symmetry of the lesions in VSBV-1 infection was seen in only 62% and not 87%. This might be due to longer observation periods in VSBV-1 cases.

The hippocampus and/or amygdala and cingulate gyrus as parts of the limbic system were the first regions affected in VSBV-1 encephalitis, accompanied or followed by involvement of the basal ganglia. Limbic encephalitis of autoimmune cause is one of the most important differential diagnoses of bornavirus encephalitis caused by either virus [2,18]. The absence of autoantibodies in limbic encephalitis cases (“seronegative” limbic encephalitis) is a strong hint for an underlying bornavirus-related etiology [2]. Of note, in horses suffering from Borna disease caused by BoDV-1, an early involvement of the limbic system has been demonstrated [19]. This is related to an olfactory portal of entry which is strongly assumed in these animals [20], as neuronal olfactory pathways directly project to the limbic system. This olfactory transmission route might also be important in humans as already suggested for both human BoDV-1 [13,17,20] and VSBV-1 infection [16]. In rat models, the spread of BoDV-1 to the brain has been effectively demonstrated by this olfactory route, and less so by subcutaneous infection ([20] and referenced therein). In VSBV-1 encephalitis, the olfactory bulb has only been tested in patient A with a PCR-cycle threshold value comparable to other brain regions in this patient [16]. Whether this infection was centripetal (as in an external route and portal of entry) or centrifugal (as in virus spreading from the brain) remained unclear. Of interest, in three of the seven VSBV-1 encephalitis patients, sinusitis was noted on first MRI examination and sinusitis was noted during first clinical consultation in two of them. It remains to be elucidated if sinusitis reflects the early entry of the virus by the olfactory route or whether this is an incidental finding. Further underscoring a possible olfactory transmission route is the finding of a mucosal T cell priming signature for VSBV-1 in a probable case (case E not included in this study as no MRI was available; [3]). In contrast, scratches and bites by VSBV-1 positive squirrels, and thus a peripheral route of infection, has also been discussed [1]. Interestingly, in a peripheral prion inoculation accident of the hand of a laboratory worker, brain MRI had shown bilateral FLAIR signals in the nuclei caudati and thalami and the patient developed variant Creutzfeldt–Jakob disease [21], highlighting the anatomical structures that are affected by peripheral pathogen inoculation.

Scarce MRI data of cerebral lesions in VSBV-1 encephalitis had already hinted at an early affection also of the basal ganglia [1–3], a finding that had later been systematically shown for BoDV-1 encephalitis [17]. In line with this data, we observed a predominance of pathologic changes in the basal ganglia in all patients, especially in the caudate nuclei, and a little less frequently in the putamen and pallidum. There was only one patient in whom basal ganglia affection was very subtle (patient A). However, this could be due to a large time gap of 70 days between second and third MR examination, so that changes in basal ganglia could have occurred and decreased in between. In cases with long disease duration and high frequency MR imaging, such as in patients D1 and D2, atrophic transformation of markedly affected basal ganglia could be observed. Hyperintensities in the basal ganglia in T1 weighted images as seen in three VSBV-1 patients can have different histopathologic correlates, such as haemorrhagic transformation and/or accumulation of lipid-rich cells. In BoDV-1 cases, an accumulation of lipid phagocytes was detected in these T1 hyperintense areas [17], in VSBV-1 both haemorrhages and accumulation of foamy cells have been described [16].

Following the limbic system and the basal ganglia, further central brain regions (such as the thalamus and the internal capsule) are affected in human VSBV-1 encephalitis, succeeded by the mesencephalon, pons and medulla oblongata, seemingly in a continuous spread. A similar pattern has been described in horses with Borna disease [19]. In contrast to the MRI findings in BoDV-1 encephalitis patients [17], three of seven patients with VSBV-1 encephalitis showed pathologic changes also in the cerebellar WM or dentate nucleus. A further spread from the pontine tegmentum through the cerebellar peduncles may be assumed in two of the three patients with cerebellar lesions in VSBV-1 infection analysed here. Viral spread could occur *per continuitatem* to directly adjacent anatomical brain regions as already speculated for human BoDV-1 encephalitis [17]. However, especially when more distant brain regions are affected during the course of disease (such as cortical regions), a spread rather along specific neural tracts seems more probable. There are numerous tracts from the thalamus to the cortex, and bornaviruses are known to travel along axonal projections [19]. Neuronal circuits between the limbic system and the thalamus do also exist which may be utilized during early stages of bornavirus encephalitis, as well as thalamo-pontine tracts which reach the brainstem and cranial nerve nuclei.

Fitting to the pattern described in this MRI study, in the four patients in whom an autopsy was performed (three patients with confirmed VSBV-1 encephalitis (cases A, B, D2) and one patient with possible VSBV-1 encephalitis (case G)), neuropathological examination had shown that most commonly the temporal and insular lobes (and thus the limbic system) as well as the basal ganglia and midbrain regions were affected [1–3,16]. Correspondingly, the highest viral loads in the confirmed cases were detected in the hippocampus, and temporal cerebral cortex, basal ganglia, and midbrain regions, the lowest in the cerebellum [16].

In two VSBV-1 patients cortical changes in the temporo-occipito-parietal region dominated on first MRI (patients B and C), which – if seen in other patients – was detected in later stages of the disease. Thus, an early cortical T2 signal increase does not seem to be part of the pathognomonic pattern but does also not rule out VSBV-1 encephalitis when present. Atrophy of markedly affected cerebral WM was seen in the single individual with long survival and imaging over a period of more than 1 year (patient D1). In accordance with findings in BoDV-1 encephalitis [17] deep WM affection occurs in a later stage of disease. Of note, our data of the number of affected brain regions and/or the extent of changes in one area do not correlate with the individual survival time and thus do thus not give hints for prognosis.

The median time until abnormalities were seen on MRI scans for 19 patients with BoDV-1 was 12 days (range 5-21; [17]). In our study of seven VSBV-1 encephalitis patients, a specification of the mean or median period of time until first pathologic imaging changes occur is not possible as the time periods between symptom onset and first and follow-up imaging were very inhomogeneous. However, congruent to abnormal BoDV-1 imaging occurrences [17], inconspicuous VSBV-1 MR scans were obtained on day two (patient G), followed by few pathologic findings on day six (patient D2), day 9 (patient A), day 23 (patient C) and day 27 (patient D1). However, as both VSBV-1 and BoDV-1 disease begin clinically unspecific, the placing of MR examinations in the exact temporal context of the respective disease is somewhat limited.

The imaging results for the two possible VSBV-1 encephalitis cases closely matched those with proven VSBV-1 etiology, as far as assessable with one and three MR images only being available within the first 14 days of disease, respectively. Thus, though still classified as “possible cases” according to the case definition [6], a VSBV-1 infection in squirrel breeder cases F and G is indeed highly likely. Of note, case F is from a non-BoDV-1-endemic area in the West of Germany, whereas case G has been living in an area with known BoDV-1 occurrence in the East of Germany and could therefore, according to the imaging pattern, also been infected with BoDV-1. Moreover, the severe MR findings in both VSBV-1 and BoDV-1 encephalitis may further help to challenge the postulation of some authors that psychiatric disease is caused by wide-spread BoDV-1 infections among humans ([22,23] for example) – a hypothesis for which several meta-analyses did not find any justification [24,25].

In this report, we show that the exact bornavirus species cannot be deduced from imaging results. Although some parameters, such as asymmetry, diffusion restriction, and involvement of cerebellar WM were different in our study of VSBV-1 encephalitis when compared to BoDV-1 encephalitis, these findings seem less important given the overall similarity of MR findings in either form of bornavirus encephalitis. Virus species discrimination is useful for prognosis as BoDV-1 disease tends to have a more rapid clinical course than VSBV-1 encephalitis [6,11]. Discrimination can so far be achieved by molecular tools and serology [6], but does not influence any possible treatment attempts however. Although the VSBV-1 encephalitis case count might seem comparably low, undiagnosed cases are to be expected in the region of natural VSBV-1 occurrence. The geographic origin of VSBV-1 is unknown, but, as several species of exotic squirrels in captivity were infected [26,27], VSBV-1 is likely to be found in the Tropics where the animals are endemic [3–5]. In tropical countries bornavirus serology and molecular assays to test for VSBV-1 in encephalitic patients will likely not be available. As MRI investigations are becoming more and more globally utilized, such imaging technologies might be helpful to readily diagnose VSBV-1 encephalitis cases also in these geographical regions. Moreover, with artificial intelligence becoming integrated in imaging studies, specific disease imaging patterns may become even better discernable. However, imaging pattern of neuroinfectious diseases (particularly in viral encephalitis) are not specific to one single disease and group of diseases. Thus, there is a certain overlap of imaging findings across different virus-mediated encephalitis entities. Regarding VSBV-1 encephalitis and the affection of parts of the limbic system, there is an overlap with e.g. HSV-1 and HHV6 encephalitis. The involvement of the basal ganglia observed in our study is also described for Japanese encephalitis, West Nile virus encephalitis and rabies encephalitis [28]. In general, for neuroinfectious diseases, we recommend using 3D T2/FLAIR, axial T2 weighted, axial DWI, and T1 weighted imaging before and after i.v. administration of contrast media.

In conclusion, we here demonstrate an imaging pattern of VSBV-1 encephalitis which closely resembles the phenotype of BoDV-1 encephalitis. The pattern is typical for bornavirus encephalitis, but non-discriminatory between the two virus species. In human encephalitis cases with the MRI phenotype shown for BoDV-1 [17] and in this study for VSBV-1, bornavirus serology and molecular testing should be performed to confirm the etiology and specify the causative bornavirus.
